# The effect of ifosfamide, epirubicin, and recombinant human endostatin therapy on a cardiac angiosarcoma: A case report

**DOI:** 10.1097/MD.0000000000015290

**Published:** 2019-04-26

**Authors:** Lijun Jiang, Xingjie Xu, Henry Davies, Kexin Shi

**Affiliations:** a The First Affiliated Hospital of Zhejiang University; b Zhejiang University, Hangzhou, Zhejiang Province, China.

**Keywords:** angiosarcoma, cardiac tumor, endostatin, epirubicin, ifosfamide, paclitaxel

## Abstract

**Rationale:**

Cardiac angiosarcoma is a rare malignant tumor, for which only surgery has been proven to be effective to date. Currently there are no reports as to whether a postoperative regimen of ifosfamide, epirubicin, and recombinant human endostatin is effective.

**Patient concern:**

The patient presented to us with chest pain and dyspnea.

**Diagnosis:**

Enhanced computerized tomography (CT) and positron emission tomography–computerized tomography (PET-CT) suggested pericarditis and an atrial perforation, but malignancy was suspected, so the patient underwent an operation to resect the tumor and repair. Pathology of the tumor reseccted at operation showed the tumor to be an angiosarcoma.

**Intervention:**

After the surgery, the patient was stared on a paclitaxel chemotherapy regimen (135 mg/m^2^ once every 3 weeks). However, 2 cycles later, pulmonary and hepatic metastases were found. Chemotherapy was then changed to ifosfamide, epirubicin (ifosfamide 2000 mg/m^2^ days 1–3, epirubicin 70 mg/m^2^ days 1–2) and recombinant human endostatin (7.5 mg/m^2^ days 1–14) in 3 weekly cycles.

**Outcome:**

Three cycles later, follow-up showed that chemotherapy had delayed progression of the pulmonary metastases, but that the hepatic node was still growing. The patient has now survived 8 months post surgery and is still on follow-up.

**Lessons:**

This case shows us that operation on late stage cardiac angiosarcomas can alleviate a patient's symptoms; postoperative paclitaxel monotherapy was insufficient and ifosfamide and epirubicin plus recombinant human endostatin has a limited effect on late stage cardiac angiosarcoma. Studies with a larger sample size are needed for verification of these findings.

## 1 Introduction

Cardiac angiosarcoma is the most common malignant cardiac tumor.^[1]^ According to existing literature, complete resection is the only proven effective treatment.^[6]^ Others such as chemotherapy or radiotherapy were effective only in sporadic cases.^[19–21]^ In this case, we aimed to discuss the effect of ifosfamide and epirubicin (IE) plus recombinant human endostatin (rh-endostatin) following palliative surgery.

## 2 Case presentation

**Figure 1:**
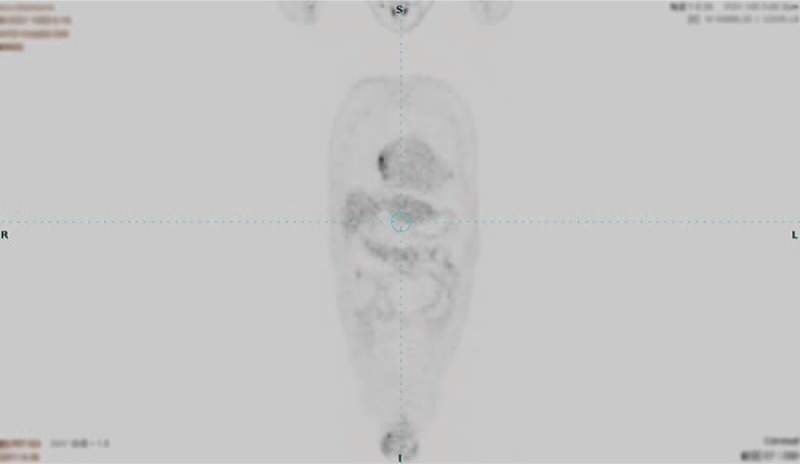
Positron emission tomography–computed tomography demonstrated increased glucose metabolism in right side of the epicardium.

**Figure 2:**
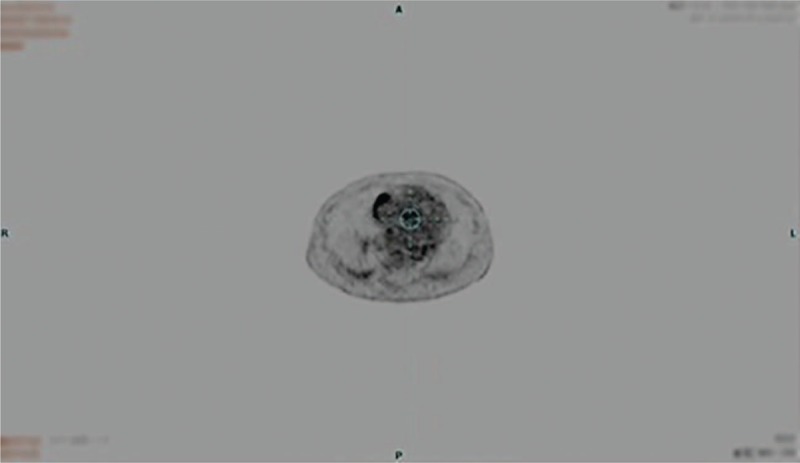
Positron emission tomography–computed tomography demonstrated increased glucose metabolism in right side of the epicardium.

**Figure 3:**
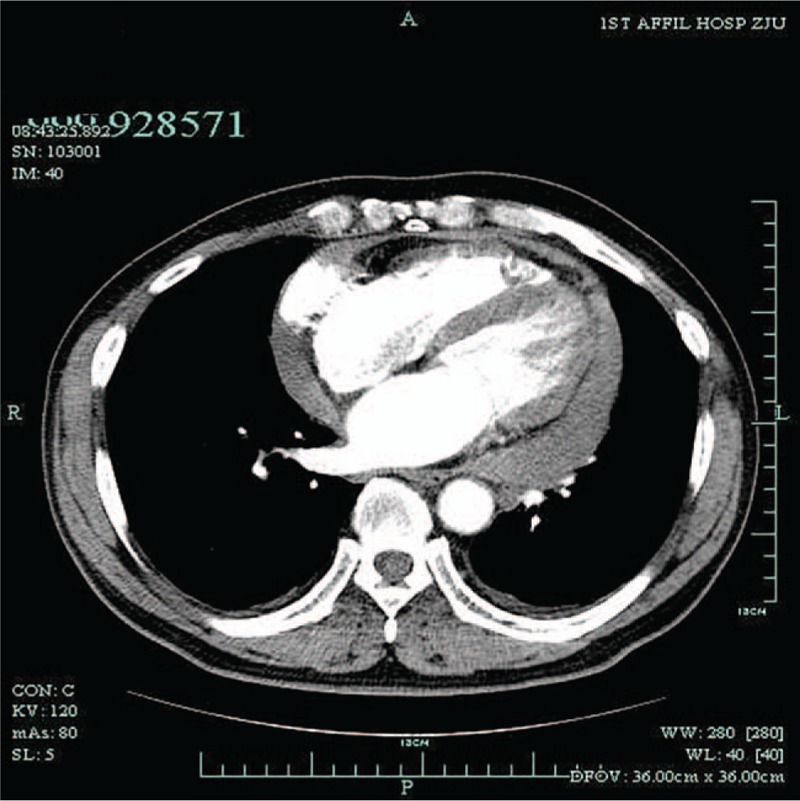
Contrast agent leaking into pericardial cavity. No obvious right atrium thickness or masses was observed.

The patient is a 61-year-old man, complaining of chest pain and dyspnea persisting for over 1 month. Medical history is nonsignificant, except for having stage 3 hypertension for 3 years, which had been well controlled with Irbesartan and dihydrochlorothiazide. Before coming to our hospital, a TTE (transthoracic echocardiogram) at a local clinic showed a 1.4-cm pericardial effusion, and a computed tomography (CT) demonstrated bilateral inflammatory effusion of lung and plural effusion. After being admitted to the local hospital, pericardialcentasis and thoracocentasis were carried out for symptom relief and diagnostic purposes. The pericardial fluid revealed nucleated cell +, red cell ++++, Rivalta test positive, CA125 (cancer antigen 125) 2013.1 IU/mL, keratin 19 13.7 ng/mL, and squamous cell carcinoma 4.2 ng/m. Pleural fluid results were Rivalta test positive, total protein 30.8 g/L, lactate dehydrogenase 238 IU/L, glucose 8.13 mmol/L, and adenosine deaminase 14 IU/L. Positron emission tomography–computerized tomography (PET-CT) revealed: increased pulmonary metabolism of bilateral posterior segments of lower lobe, inflammatory effusion considered; upper lobe emphysema, pleural effusion, and a right lower lobe node, considered to be hyperplasia; increased metabolism of right epicardium, inflammation considered (Figs. 1 and 2). Other tests, including T-SPOT, myeloperoxidase, proteinase 3, and antineutrophil cytoplasmic antibodies, were all negative, except for increased CA125 (140.1 IU/mL) in the peripheral blood. Ten days after being discharged from hospital, the patient experienced a syncope episode, so came to our hospital for further diagnosis and treatment. After an enhanced-CT (Fig. 3) reporting contrast agent entered into pericardial cavity and an echo finding multiple lymph node enlargement, including a 1.1 × 0.6 cm left superclavicular node, a 1.9 × 0.7 cm left axillary node and a 3.0 × 1.1 cm right axillary node, an MDT meeting came to the conclusion that it was reasonable to suspect cardiac tumor, most likely a cardiac sarcoma, and surgery was recommended.

**Figure 4:**
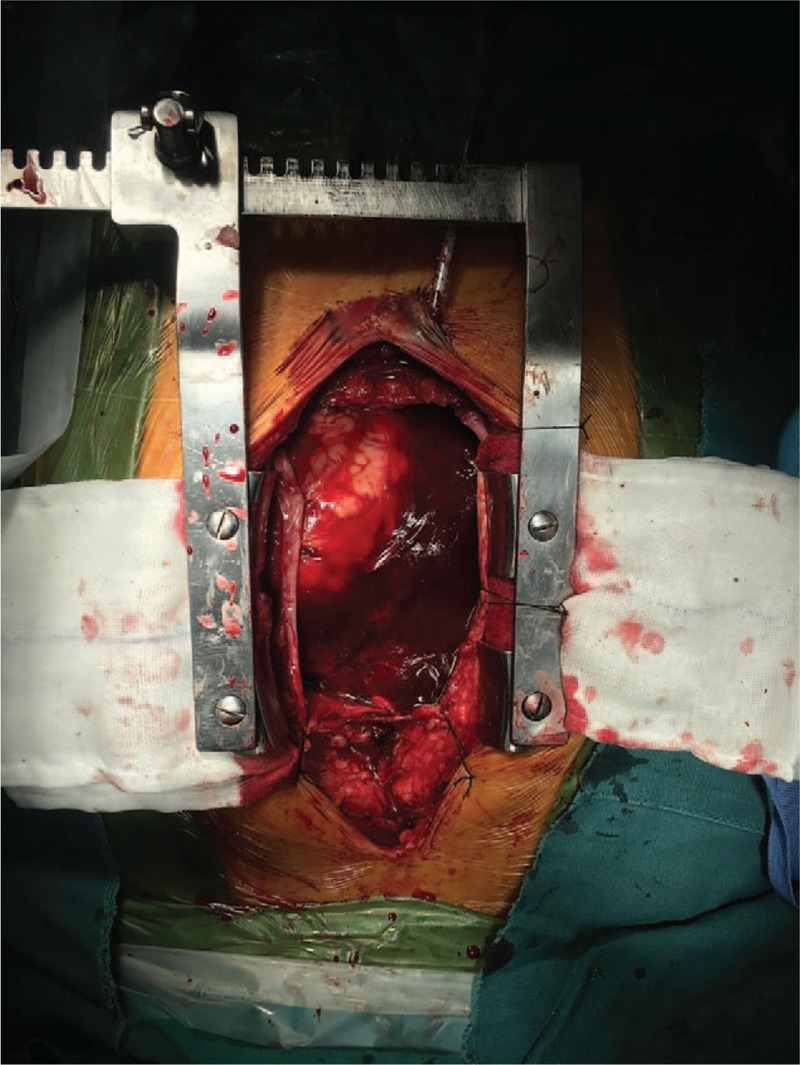
Pericardial sac and blood clot.

**Figure 5:**
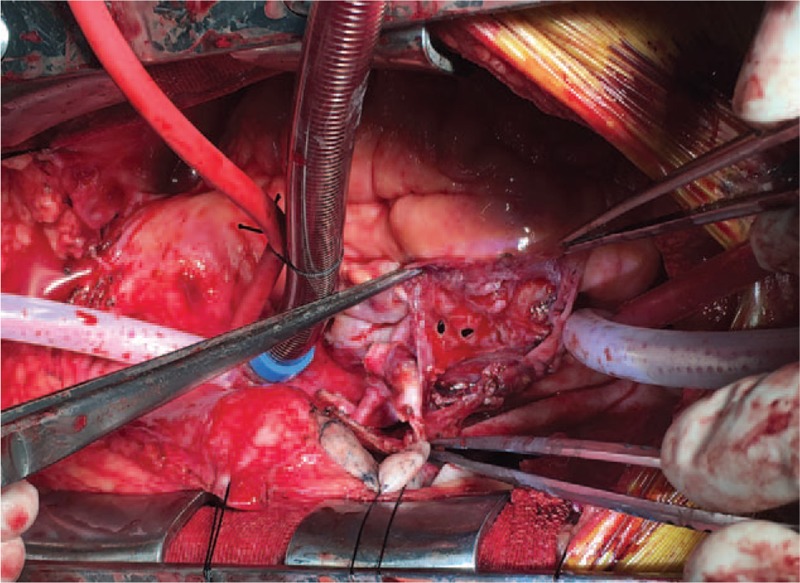
Atrial perforation adjacent to the interatrial septum.

The ethical approval and consent was checked by ethical department of the 1st affiliated hospital of Zhejiang University. The surgery was ordered on the 2nd day of admission. A mediasternotomy and cardiopulmonary bypass through the femoral artery and vein were chosen. When the pericardial cavity was opened, abundant blood clots were seen (Fig. 4). After cleaning of the clots, a perforation of the right atrium, adjacent to interatrial septum, and a thickened atrium wall surrounding the hole were observed (Fig. 5). Part of the affected atrium and pericardium were resected; however, some neoplastic tissue still remained on the tricuspid valve ring. The right atrium was then reconstructed with a bovine pericardium. The pathology report of the frozen section was benign cyst with inflammatory cell infiltration. And postoperative recovery was uneventful. One week later, immunohistopathology reported spindle shaped heterocyst and focal vascular like lacuna formation with local necrosis, CD34(+), CD31(+), Fil-1(+), KI67(30%+), F8-R-Ag(+), CK(−), SMA(−), Desmin(−), P53(−), S100(−), considering angiosarcoma. One month later from the surgery, the patient was then started on a paclitaxel chemotherapy regimen (135 mg/m^2^ once every 3 weeks). After 2 cycles, a CT scan showed enlarged pulmonary nodes and hepatic metastases were found, the chemotherapy regimen was changed to IE (ifosfamide 2000 mg/m^2^ days 1–3, epirubicin 70 mg/m^2^ days 1–2) + recombinant human endostatin (7.5 mg/m^2^ days 1–14), in 3 weekly cycles. After 3 cycles, primary focus and lung metastases were stable without progression, but hepatic metastases were still enlarging. It is now 8 months postsurgery and the patient is still attending follow-up. The patient has provided informed consent for publication of the case.

## 3 Discussion

Primary cardiac tumors are rare with a prevalence of 0.02%, amongst which 25% are found to be malignant by pathology. Cardiac angiosarcomas are the most common histopathologic type originating from vascular or lymphatic endothelial cells, accounting for 30% of cardiac malignant tumors.^[1]^ The most common presentation for angiosarcoma is one of chest tightness and pain, as in our patient. When the tumor infiltrates the cardiac wall, necrosis, perforation, and increased vascular permeability can cause pericardial effusion, even cardiac tamponade and its subsequent symptoms.^[2,3]^ Another reason for symptom occurrence is tumor bulk. When the tumor grows to a certain size, it can cause blood flow obstruction, valvular stenosis, or insufficiency. Other symptoms include cardiac arrhythmia and pulmonary embolism.^[4]^ Usually when patients feel discomfort, the tumors are of a certain size and distal metastases may have already developed. The most common location of distal metastasis is lung, but liver, spleen, bone, and brain have also been reported.

As the symptoms are nonspecific, diagnosis of cardiac angiosarcoma needs advanced testing like magnetic resonance imaging (MRI), CT, ultrasonography, PET-CT, and autopsy. Cardiac MRI and CT scans can demonstrate tumor location, size, and local invasion.^[5]^ Contrast studies are also helpful in differentiating thrombus, myxomas, and infiltrative growth. TTE and TEE are the most common diagnostic methods of cardiac disease, as they are able to show tumor flexibility, mobility, and attachment width dynamically.^[6]^ Before surgery or autopsy, some characteristics may indicate malignancy; right atrium mass; an enhanced CT or MRI demonstrating necrosis, and an abundant blood supply within the mass; a broad base or poorly defined attachment and myocardial invasion.^[7]^ PET-CT/MRI is a novel diagnostic technique, combining functional imaging with anatomic imaging, showing tissue metabolic activity of a certain part of body. It is helpful in mass differential diagnosis, surgical planning, cancer staging, and radiotherapy. In malignant masses, the maximum standardized uptake value (SUVmax) is higher than nonmalignant mass.^[8]^ In this case, the discrepancy between PET-CT and ultimate diagnosis in location of neoplasm came from the proximity of the 2 layers of pericardium and pitfall resolution of PET-CT. Definitive diagnosis of cardiac angiosarcoma depends on the tissue biopsy resected in surgery or fine-needle aspiration. Histopathologic characteristics are vasoformative features including cytoplasmic vacuoles containing red blood cells, hemophagocytosis and endothelial wrapping. Immunohistochemical staining of cardiac angiosarcomas is always CD34, CD31, FKI-1, and von Willebrand factor positive.^[9]^ When planning for surgery or other treatment strategies, it is necessary to take all these test results into consideration.

Due to the rarity of cardiac angiosarcomas, only a few gene mutations have been reported so far, these include KDR (G618R), MDM4,^[10]^ POT1 gene missense mutation in TP53-negative Li-Fraumeni-like family,^[11]^ numerical and structural changes in the chromosomes and the p53 gene.^[12]^ KDR, also called VEGFR-2, plays an important role in angiogenesis and vasculogenesis. Current therapies focusing on this target include fruquintinib, lenvatinib, pazopanib, regorafenib, vatalanib, and tivozanib. Only pazopanib has been applied to a case on a patient with metastatic cardiac angiosarcoma and resulted in the distal metastasis’ complete remission.^[13]^ No target drug has been found for the other mutations. For the gene mutations run on our patient, NRAS (2,3,4 exon mutation), EGFR (18,19,20,21 exon mutation), KRAS (2,3,4 exon mutation), BRAF (V600E mutation), ROS1 fusion, and EML4-ALK fusion were all negative.

Prognosis of primary cardiac sarcoma is especially poor for angiosarcomas. According to the 4 existing case series, survival time is 7, 5, 26.6, and 13 months.^[14–17]^ When compared to other histopathology types, primary angiosarcoma has a shorter survival time (5 vs 17 months). Distal metastasis is an important predictor of poor prognosis, as the 3rd case series reported, with a survival time of just 5 months compared to 15 months in a localized patient, and the 4th case series which showed an overall survival of 6 months for angiosarcomas with distal metastases compared to 19.5 months for localized ones. So far, surgical resection is the only treatment method proved to reduce local tumor mass and alleviate obstruction symptoms such as peripheral edema, chest pain, and dyspnea. As the 3rd and 4th case reports revealed, surgical resection of a cardiac tumor mass can improve survival time from 6 to 17 months and 5 to 17 months, respectively. Though there is no statistical evidence, it is reasonable to believe that the prognosis of R0 resection (absence of macroscopic and microscopic evidence of tumor in pathological report) is better than R1 (microscopic residual) and R2 (macroscopic residual).

Paclitaxel is a chemotherapy drug targeting tubulin which has been used effectively in angiosarcoma.^[18]^ There are 3 cases reported of treating cardiac angiosarcoma with paclitaxel to good effect, from partial response to complete remission of primary tumor and distal metastasis but unfortunately it was not work in our patient.^[19–21]^ Ifosfamide is a broad used chemotherapy drug in angiosarcoma, combining with doxorubicin achieved a well effect was reported and epirubicin, another anthracycline drug, always combined with ifosfamide as chemotherapy regimen for soft-tissue sarcoma.^[22]^ Endostatin is a wide spectrum tumor angiogenesis inhibitor, with slight side-effects.^[23]^ As angiosarcomas originate from endothelial cells, it is reasonable to think it may work in this patient, though there was no clinical trial or cases reported, and it turned out the effect is limited.

Reviewing this case, it was a dilemma whether to choose surgery or not even when malignant cardiac tumor is suspected. According to the report, the operation undoubtedly can improve survival time, especially for R0 resection, but not in R1 or R2 resection because it can delay the chemotherapy or radiotherapy and cause tumor cell spreading through blood stream. But acute cardiac tamponade can result at any time once the tumor is of sufficient size, so even though circulation blockage was slight and distal metastasis were suspected, surgery was necessary for this patient. Furthermore, it we would not have been able to start the aggressive chemotherapy regimen without have positive tissue pathology.

## 4 Conclusion

Cardiac angiosarcoma is a rare but aggressive malignant tumor with poor prognosis. Early diagnosis and treatment can improve a patient's prognosis markedly; however, early metastasis are also common, so it is necessary to evaluate the stage of the cancer, local invasion and distal metastasis to decide on an appropriate therapeutic strategy. For cardiac angiosarcomas with distal metastases or those considered locally unresectable, surgery should still be considered as it has the potential to alleviate symptoms and possibly prevent cardiac tamponade. Postoperative paclitaxel monotherapy was ineffective and IE plus rh-endostatin has a limited effect on late stage cardiac angiosarcoma. Further clinical research of larger numbers of patients is needed.

## Author contributions

Lijun Jiang, surgeon of the 1st affiliated hospital of Zhejiang University, cardiac surgery doctor of Peking Union Medical College, visiting scholar of Stanford University School of Medicine.

**Conceptualization:** Lijun Jiang.

**Data curation:** Kexin Shi.

**Resources:** Lijun Jiang.

**Supervision:** Lijun Jiang.

**Writing – original draft:** Xingjie Xu.

**Writing – review & editing:** Xingjie Xu, Henry Davies.
